# Love Amongst the Wise Men of the East

**Published:** 1902-09

**Authors:** 


### Love Amongst the Wise Men of the East.

In the August number of *The Louisville Monthly Journal of Medicine and Surgery,* Dr. Joseph M. Mathews, former President of the American Medical Association, and of a half dozen other large organizations, and President of the State Board of Health of Kentucky, in an editorial correspondence from New York City, has the following:

"The moral of all this is, doctor, take a rest, and don't take life too seriously. The latter part of the above sentence is a quotation from a remark so often made by the brilliant editor of *The Medical Mirror,* Dr. I. N. Love. By the way, that reminds me of his wonderful success here in New York, of which I have had a personal demonstration in the last few days. One would think that perhaps the most difficult feat to accomplish would be for a doctor to rush immediately into business here among the 'wise men of the East.' But this Dr. Love has done. In common parlance, he has 'caught on' and you cannot 'down him.' But this is not to be wondered at when we consider the extensive acquaintance he has with the medical profession throughout the State. I am not overstating it in saying that he is the best-known man in the medical profession in America. As I watched him a few days ago dispatch business I thought of what could be accomplished by some one of the large life insurance companies here if they could secure his services as referee. A personal acquaintance with thousands of doctors, and a capacity for work scarcely equaled—think what he could do in this line.

"But I do not intend to allure doctors away from their homes and cause a stampede to the East by reciting this good fortune of Dr. Love. Far from it, for my observation of things medical here has taught me that the average doctor has a 'hard road to travel.' But outside of the matter of 'bread and butter' this strenuous life led by the New York doctor is a killing one."

